# Successful resumption of tocilizumab for rheumatoid arthritis after resection of a pulmonary *Mycobacterium avium* complex lesion: a case report

**DOI:** 10.1186/s12890-015-0130-z

**Published:** 2015-10-23

**Authors:** Ho Namkoong, Sadatomo Tasaka, Mitsuhiro Akiyama, Kazuma Yagi, Makoto Ishii, Katsuya Suzuki, Mitsutomo Kohno, Naoki Hasegawa, Tsutomu Takeuchi, Tomoko Betsuyaku

**Affiliations:** Division of Pulmonary Medicine, Department of Medicine, Keio University School of Medicine, 35 Shinanomachi, Shinjuku-ku, Tokyo, 160-8582 Japan; Division of Rheumatology, Department of Medicine, Keio University School of Medicine, Tokyo, Japan; Division of General Thoracic Surgery, Keio University School of Medicine, Tokyo, Japan; Center for Infectious Diseases and Infection Control, Keio University School of Medicine, Tokyo, Japan

**Keywords:** Biological agents, *Mycobacterium avium* complex (MAC), Resection, Rheumatoid arthritis, Tocilizumab

## Abstract

**Background:**

Biological agents inhibiting TNF-α and other molecules involved in inflammatory cascade have been increasingly used to treat rheumatoid arthritis (RA). However, it remains controversial whether biological agents can be used safely in a patient with an underlying chronic infectious disease.

**Case presentation:**

A 63-year-old woman who had been treated with tocilizumab (TCZ), anti-interleukin-6 receptor antibody, for RA presented to our outpatient clinic due to hemoptysis. She was diagnosed with pulmonary *Mycobacterium avium* complex (MAC) infection, and high-resolution computed tomography (HRCT) showed a single cavitary lesion in the right upper lobe. After diagnosis of pulmonary MAC disease, TCZ was discontinued and combination chemotherapy with clarithromycin, rifampicin, ethambutol and amikacin was started for MAC pulmonary disease. Since the lesion was limited in the right upper lobe as a single cavity formation, she underwent right upper lobectomy. As her RA symptoms were deteriorated around the operation, TCZ was resumed. After resumption of TCZ, her RA symptoms improved and a recurrence of pulmonary MAC infection has not been observed for more than 1 year.

**Conclusion:**

This case suggested that TCZ could be safely reintroduced after the resection of a pulmonary MAC lesion. Although the use of biological agents is generally contraindicated in patients with pulmonary MAC disease, especially in those with a fibrocavitary lesion, a multimodality intervention for MAC including both medical and surgical approaches may enable introduction or resumption of biological agents.

## Background

Various types of biological agents such as infliximab and tocilizumab (TCZ) have been increasingly used to treat rheumatoid arthritis (RA) because of their effectiveness [1, 2]. RA patients are often complicated by pulmonary lesion including interstitial pneumonia and bronchiectasis that is vulnerable to infection [3, 4]. According to the recent systematic review, both standard-dose and high-dose biological agents are associated with the increased risk of serious infections, compared with traditional disease-modifying anti-rheumatic drugs (DMARDs) [5]. With respect to the difference in susceptibility between the classes of biologics, no difference in the risk of infection has been reported between TCZ and others, although the Cochrane review in 2011 reported that abatacept, cytotoxic T lymphocyte antigen 4-immunoglobulin, was significantly less likely to cause infection than infliximab and TCZ [6]. Moreover, it has been shown that biological agents are associated with a significant increase in mycobacterial diseases [7]. Concerning the types of mycobacterial diseases, Winthrop and coworkers reported that nontuberculous mycobacteria (NTM) infections were more common than tuberculosis among patients receiving biologics [8]. Especially in Japan, the most recent nationwide survey revealed that the incidence rate of pulmonary NTM disease (14.7 persons per 100,000 person-years) may exceed that of tuberculosis in general population, and that Japan may have one of the highest incidence rates of pulmonary NTM disease worldwide [9]. Whereas tuberculosis can usually be controlled by the standard chemotherapy, no effective chemotherapy has been established against *Mycobacterium avium* complex (MAC), leading to aggravation of MAC infection during immunosuppressive therapy [10, 11]. According to Japanese postmarketing surveillance of TCZ in RA patients, the incidence of NTM infections (0.22 %) is higher than that of tuberculosis (0.05 %) [12]. Although many of RA patients have underlying pulmonary lesions and other risk factors for potential NTM infection, it is still controversial whether biological agents can be a risk of exacerbation of pre-existing pulmonary NTM disease [11]. Consequently, a strategy for the management of NTM in RA patients subjected to treatment with biologics remains to be established.

In this report, a case of pulmonary MAC disease in an RA patient who successfully resumed TCZ after the resection of a single cavitary lesion is presented. Although the use of biological agents is generally contraindicated in patients with pulmonary MAC disease, especially in those with a fibrocavitary lesion, a multimodality approach for MAC may enable introduction or resumption of biological agents. This report is in compliance with the Helsinki Declaration.

## Case presentation

**Fig. 1:**
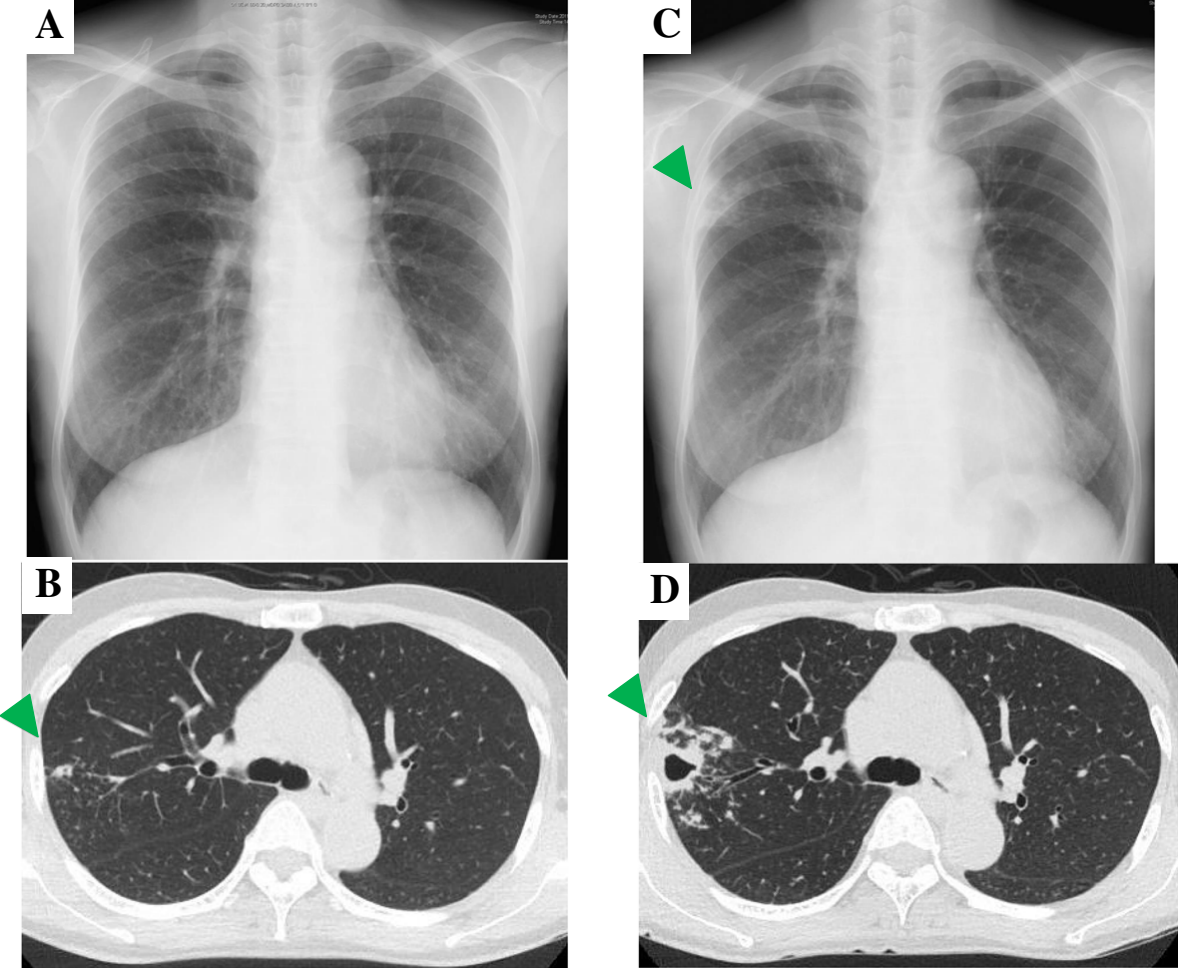
Serial changes on chest X-ray and chest computed tomography findings. **a** Chest X-ray taken immediately before starting tocilizumab (TCZ), showing subtle nodular infiltrates. **b** CT scan taken immediately before starting TCZ, showing a small nodular shadow in the right upper lobe (*arrowhead*). **c** Chest X-ray taken when the patient developed hemoptysis 2 years after starting TCZ, showing infiltration and cavity formation in the right upper lung field (*arrowhead*). **d** CT scan taken when the patient developed hemoptysis 2 years after starting TCZ, showing consolidation, cavity formation, bronchiectasis, and centrilobular nodules in the right upper lobe (*arrowhead*)

In September 2013, a 63-year-old woman was referred to our outpatient clinic due to hemoptysis and a pulmonary lesion on high-resolution computed tomography (HRCT). Her height was 165.0 cm and body weight was 46.0 kg. The patient never smoked but had a medical history of Crohn’s disease, which remained in remission, and RA that was diagnosed in 2010 according to the criteria of the American College of Rheumatology. She had been treated with prednisolone (PSL) (5 mg/day) and methotrexate (12 mg/week). Because the disease activity was not properly controlled with these medications, methotrexate was stopped and 360 mg of TCZ was administered intravenously once every 4 weeks from October 2011. At this time, the visual analogue scale (VAS) was 37 mm and the disease activity score (DAS) 28–C-reactive protein (CRP) was 3.81. When TCZ was introduced, her chest radiograph was normal (Fig. 1a), but HRCT showed a small nodular shadow in the right upper lobe of the lung (Fig. 1b). Although the patient had no respiratory symptoms with no pathogenic bacteria isolated from the sputum, she was prescribed 400 mg/day clarithromycin (CAM) as a monotherapy before her referral to our department. Two years after the initiation of TCZ, she was admitted for hemoptysis, and a chest radiograph showed infiltration and cavity formation in the right upper lobe (Fig. 1c). HRCT also showed consolidation, cavity formation, bronchiectasis, and centrilobular nodules in the right upper lobe (Fig. 1d). When admitted, her body temperature was 36.4 °C. Coarse crackles were auscultated over the right upper lung field and joint pain was positive in her left wrist, right elbow, and metatarsophalangeal joints of the right third and fourth toes. There were no abnormal findings on complete blood counts and biochemistry tests except for mild leukocytopenia (white blood cells, 3300/μL) (Table 1). The anti-glycopeptidolipid core IgA antibody was positive (2.44 U/mL), and the QuantiFERON® TB Gold test was negative. Pulmonary MAC disease was diagnosed because the sputum culture was positive for MAC twice. A Broth MIC® NTM showed that the isolated MAC was sensitive to CAM (minimum inhibitory concentration, 1 μg/mL) despite 2-year monotherapy with CAM. Combination chemotherapy with 800 mg/day of CAM, 450 mg/day of rifampicin (RFP), 500 mg/day of ethambutol (EB), and thrice weekly intravenous amikacin (600 mg/per dose) were started. Because TCZ might have contributed to the exacerbation of the pulmonary MAC disease, TCZ therapy was discontinued after the diagnosis of pulmonary MAC disease. In addition, PSL was tapered to 2 mg/day, and nonsteroidal anti-inflammatory drugs were started for RA. At this time, the disease activity of her RA was relatively stable (VAS, 6 mm; DAS 28-CRP, 1.99).

**Table 1:** Laboratory findings on admission

Complete blood count	
White blood cells	3300/μL
Band cells + Seg cells	54.1 %
Lymphocytes	32.7 %
Monocytes	8.1 %
Eosinophil granulocytes	4.5 %
Basophil granulocytes	0.6 %
Hemoglobin	13.8 g/dL
Mean corpuscular volume	94/fL
Platelets	182 × 10^3^ /μL
Biochemistry	
Total protein	6.4 g/dL
Albumin	4.2 g/dL
Total bilirubin	0.7 mg/dL
Glutamic oxaloacetic transaminase	20 IU/L
Glutamic pyruvic transaminase	14 IU/L
Lactate dehydrogenase	180 IU/L
Urea nitrogen	11.2 mg/dL
Creatinine	0.64 mg/dL
Sodium	143.2 mEq/L
Potassium	3.9 mEq/L
Chloride	109 mEq/L
Alkaline phosphatase	197 IU/L
Gamma-glutamyl transferase	13 IU/L
Serological studies	
C-reactive protein	0.01 mg/dL
Matrix metalloproteinase 3	42.2 ng/mL
β-D-glucan	<3.0 pg/mL
*Aspergillus* antigen	0.0 COI
*Cryptococcus* antigen	0.0 COI
QuantiFERON® TB Gold test	Negative
Anti-glycopeptidolipid core IgA antibody	2.44 U/mL

**Fig. 2:**
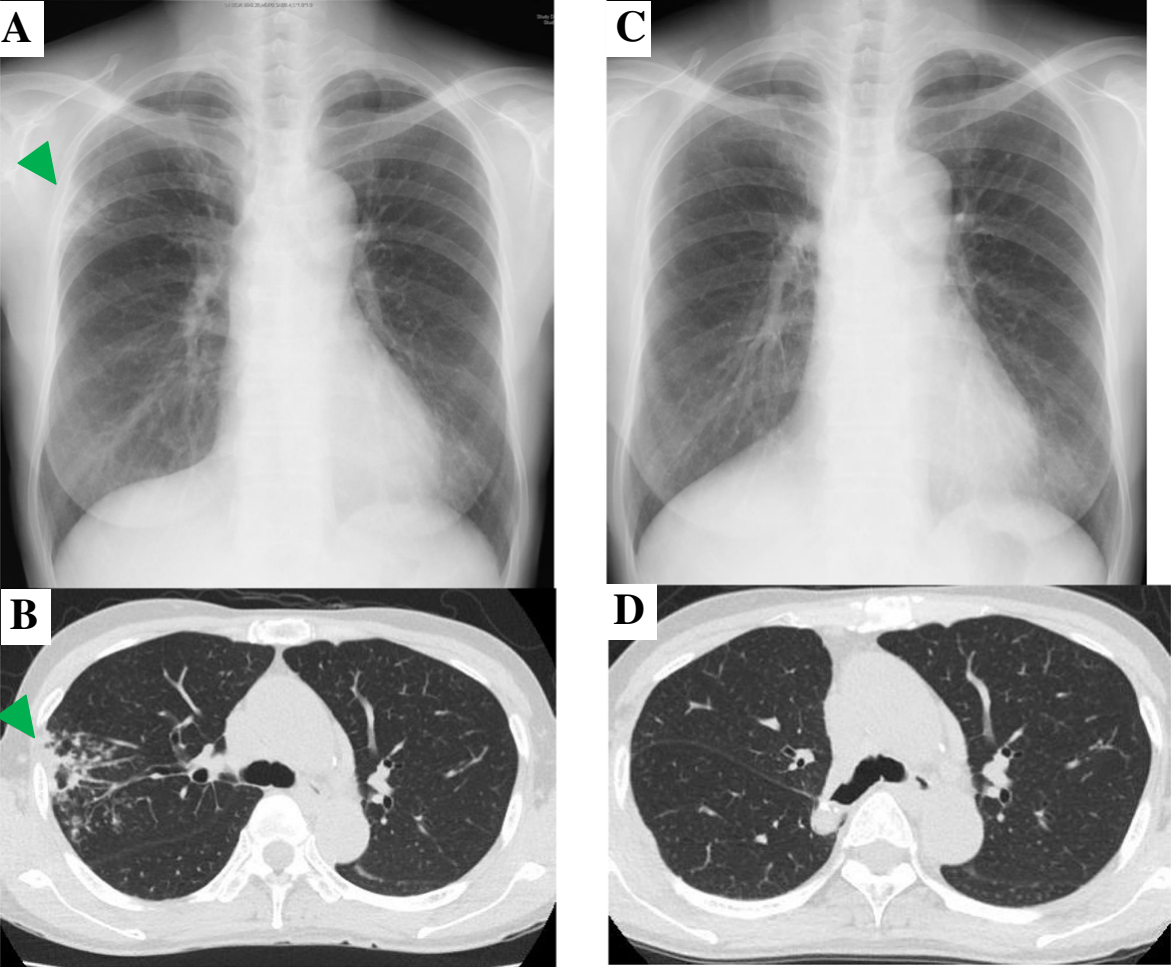
Serial changes on chest X-ray and chest computed tomography findings. **a** Chest X-ray taken 3 months after chemotherapy against *Mycobacterium avium* complex (MAC), showing improved infiltration (*arrowhead*). **b** CT scan taken 3 months after chemotherapy against MAC, showing diminished consolidation and centrilobular nodules in the right upper lobe (*arrowhead*). **c** Chest X-ray taken 1 year after resuming TCZ, showing no abnormal findings other than the postoperative findings after the right upper lobectomy. **d** CT scan taken 1 year after resuming TCZ, showing no postoperative findings of right upper lobectomy

**Fig. 3:**
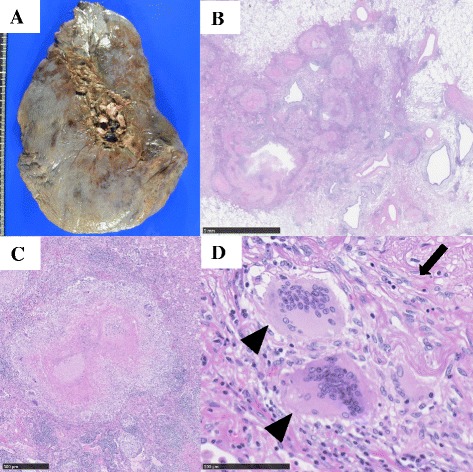
Photograph and photomicrographs of the lung. **a** Photograph of a cross-sectional specimen from the resected right upper lung. **b** Photomicrographs showing an epithelioid granuloma with necrosis (bar, 5 mm). **c** Photomicrographs showing an epithelioid granuloma with necrosis (bar, 500 μm). **d** Photomicrograph showing Langhans giant cells (*arrowheads*) and epithelioid cells (*arrow*) (bar, 100 μm)

Although 3 months of anti-MAC treatment improved the consolidation and centrilobular nodules, the cavitary lesion and bronchiectasis were still significant on chest X-ray and HRCT (Fig. 2a, b). Her right upper lobe was resected (Fig. 3), since her lesion was limited to a single lobe and the cavitary lesion seemed refractory to drug therapy. As expected, tissue culture of the resected specimen tested positive for MAC. Although her pulmonary MAC lesion was totally removed with the operation and microbiological examinations remained negative, CAM, RFP, and EB were continued to prevent a relapse of MAC pulmonary disease. Because she reported deterioration of her joint symptoms around the time of the operation (VAS, 38 mm; DAS 28-CRP, 4.07), TCZ was resumed 1 month after resection.

Two months after the restarting of TCZ, her joint symptoms had totally improved; the VAS improved from 38 to 3 mm, and DAS 28-CRP improved from 4.07 to 1.06. In terms of the pulmonary MAC disease, no new lesion was found by HRCT at 1 year after the resumption of TCZ (Fig. 2c, d).

## Conclusions

MAC is one of the most common opportunistic pathogens in RA patients on biological agents [13]. Although most immunocompetent patients with MAC infection maintain a stable clinical course for years, immunocompromised patients often show rapid progression of the disease. Previous MAC infections in RA patients are a particularly controversial issue, because the aggravation of MAC infection requires abandoning effective treatment with biological agents [11, 14]. However, some case reports and the new joint statement published by the Japanese Respiratory Society (JRS), Japan College of Rheumatology (JCR), and Japanese Society for Tuberculosis (JSTB) in 2014 proposed that, in certain RA patients with pulmonary MAC disease, biological agents can be safely used in combination with chemotherapy for pulmonary MAC disease [15, 16]. In the case series by Mori and colleagues, anti-TNF agents could be safely reintroduced in seven patients without exacerbation of the MAC infection [14]. Nakahara and coworkers also described a case of successful reintroduction of TCZ without aggravation of MAC infection under the standard chemotherapy [15]. However, there has been no report of surgical resection of MAC lesion followed by successful reintroduction of biologics. In this regard, this is the first case report which indicates that biological agents might be safely resumed after surgery.

In this case, HRCT taken before the initiation of TCZ treatment showed small nodular opacity in the right upper lobe, suggesting pre-existing MAC disease. Since monotherapy with macrolides can induce macrolide-resistance, it is not an appropriate treatment in the presence of MAC disease [17]. Therefore, bronchoscopy should have been performed to make a diagnosis of pulmonary MAC disease so that combination chemotherapy could be introduced at an earlier time point.

After the exacerbation of pulmonary MAC disease, HRCT showed consolidation, cavity formation, bronchiectasis, and centrilobular nodules that were localized only in the right upper lobe. According to the JSTB statement about the resection of pulmonary NTM disease, a single cavity lesion is a good candidate for surgery to suppress the activity of pulmonary NTM disease [18]. From this standpoint, our approach to eradicating MAC lesions was appropriate.

According to the recent joint statement, patients with NTM are in principle prohibited from using biological agents [18]. The statement also mentioned that using biological agents could be considered with full evaluation of the risk and benefit, only in the following cases: (i) the causative pathogen is MAC; (ii) the radiographic features are of the nodular/bronchiectatic type; (iii) the existing pulmonary lesion is limited; (iv) the patient’s general performance status is good; (v) chemotherapy against NTM could be given in the long term with a good treatment response; and (vi) biological agents are strongly needed because of the high disease activity of RA. It also stated that using biologics in those with a fibrocavitary lesion of NTM is a contraindication [18]. The present case indicated the possibility of safe reintroduction of biological agents after resection, even in cases with a fibrocavitary lesion, when the cavitary lesion is localized in a single lobe. Since no difference in the risk of infection has been reported between TCZ and other biological agents [7], we considered that this strategy might be applied not only for TCZ but also for other biologics.

One of the discussion points is the validity of surgical resection against MAC disease. In this case, her hemoptysis disappeared after 3 months of chemotherapy and the infiltrates around the cavity were also resolved. However, the cavitary lesion, which could discharge mycobacteria and might predispose the patient to later recurrence, was not changed. In general, the cavitary lesion of MAC patients is difficult to be resolved by chemotherapy alone and is good indication for surgical resection [18, 19]. Especially for this patient with high disease activity of RA, we thought it reasonable to remove the cavitary lesion surgically in order to resume biological agents.

Another discussion point is the duration of concurrent chemotherapy against MAC as well as perioperative chemotherapy. It was possible to discontinue the chemotherapy against MAC when the MAC lesion was totally removed by surgery. However, it was assumed that chemotherapy should be continued to prevent the growth of minimal MAC lesion, which was undetectable by HRCT. A retrospective review of pulmonary resection in patients with NTM showed that postoperative chemotherapy might contribute to decreasing the relapse rate [20]. The JSTB statement about the resection of pulmonary NTM disease also recommended postoperative adjuvant chemotherapy [18]. In any case, careful follow-up to monitor the re-emergence of the MAC lesion or other opportunistic infections is needed as long as the patient is receiving biological agents.

In conclusion, an RA case for which TCZ was safely reintroduced after resection of the pulmonary MAC lesion was presented. Although the use of biological agents is generally contraindicated in patients with pulmonary MAC disease, especially with a fibrocavitary lesion, a multimodality approach for MAC may be considered in order to use biological agents safely.

## Consent

Written informed consent was obtained from the patient for publication of this case report and accompanying images. A copy of the written consent is available for review by the Editor of this journal.

## Abbreviations

RA: Rheumatoid arthritis
TCZ: Tocilizumab
MAC: Mycobacterium avium complex
HRCT: High-resolution computed tomography
DMARDs: Disease-modifying anti-rheumatic drugs
NTM: Nontuberculous mycobacteria
PSL: Prednisolone
VAS: Visual analogue scale
DAS: Disease activity score
CRP: C-reactive protein
CAM: Clarithromycin
RFP: Rifampicin
EB: Ethambutol
JRS: Japanese Respiratory Society
JCR: Japan College of Rheumatology
JSTB: Japanese Society for Tuberculosis
